# HEALTH BEHAVIORS AND PSYCHOLOGICAL WELL-BEING ALLEVIATE DIABETES-RELATED DISABILITY DETERIORATION

**DOI:** 10.1093/geroni/igz038.302

**Published:** 2019-11-08

**Authors:** Yi-Hsuan Tsai, Ching-Ju Chiu

**Affiliations:** Institute of Gerontology, National Cheng Kung University, Tainan, Taiwan

## Abstract

Diabetes is known to increase the risk of disability, which may be buffered by health behaviors and psychological factors. However, few existing studies examine how these factors affect disability in diabetic patients over time. The present study assessed the extent to which diabetes affected disability with age and how that effects differed by health behaviors and psychological well-being in older Taiwanese. The data of 5131 adults aged 50 and older were drawn from the 1996-2007 Taiwan Longitudinal Study on Aging. A cohort sequential multilevel modeling was employed to explore the effects of sociodemographic, comorbidities, lifestyle, and psychosocial factors in mediating and moderating the link between diabetes and disability. Disability was measured by mobility limitation in 1999, 2003, and 2007, while health behaviors and psychological factors were measured in 1996, 1999, and 2003 to be lagged time-varying covariates in random effect model analyses. Our results showed that adults with diabetes had more mobility limitation (β(diabetes) =3.031, P<.001) and progressed each year with ageing (β(diabetes*age) =0.061, P<.005). Exercising more than four times a week reduced the risk of disability by 51 % in diabetic patients (β(diabetes*exercise≥4 times) =-1.220, P<0.05). Social participation (β(social participation)=-0.631, P<.005), stress (β(stress)=0.651, P<.001) and depression (β(depression)=0.144, P<.001) had independent effects on the risk of disability in adults, but the interaction with diabetes was not significant. To conclude, exercise is the most powerful factor to alleviate the risk of disability in diabetic patients.

